# Impact of cardiometabolic comorbidities on clinical characteristics, prescription patterns and retention rate of first b/tsDMARD treatment in 5299 European real-world patients with psoriatic arthritis

**DOI:** 10.1136/rmdopen-2025-006477

**Published:** 2026-05-07

**Authors:** Zohra Faizy Ahmadzay, Lykke Midtbøll Ørnbjerg, Mikkel Østergaard, Kasper Yde Jensen, Jacob Brauner Jørgensen, Jette Heberg, Anne Gitte Loft, Brigitte Michelsen, Gareth T Jones, Pasoon Hellamand, Signe Møller-Bisgaard, Mehrdad Shoae Kazemi, Parham Karimi Reikandeh, Jakub Závada, Pavel Horák, Miguel Bernardes, Elsa Vieira-Sousa, Isabel Castrejón, Lucía Otero-Varela, Catalin Codreanu, Laura Kuusalo, Vappu Rantalaiho, Anne C Regierer, Andreas Reich, Burkhard Möller, Raphael Micheroli, Pawel Mielnik, Sella Aarrestad Provan, Karin Lass, Sigrid Vorobjov, Ziga Rotar, Katja Perdan Pirkmajer, Florenzo Iannone, Fabrizio Conti, Bjorn Gudbjornsson, Daniela Di Giuseppe, Marleen van de Sande, Gary J Macfarlane, Handan Yarkan-Tuğsal, Bente Glintborg, Merete Lund Hetland

**Affiliations:** 1 Copenhagen Center for Arthritis Research (COPECARE), Rigshospitalet Center for Rheumatology and Spine Diseases, Glostrup, Denmark; 2 Department of Clinical Medicine, University of Copenhagen, Copenhagen, Denmark; 3 Department of Rheumatology and DANBIO, Aarhus University Hospital, Aarhus, Denmark; 4 Department of Clinical Medicine, Aarhus University, Aarhus, Denmark; 5 Center for Treatment of Rheumatic and Musculoskeletal Diseases (REMEDY), Diakonhjemmet Hospital, Oslo, Norway; 6 Research Unit, Sørlandet Hospital, Kristiansand, Norway; 7 Aberdeen Centre for Arthritis and Musculoskeletal Health (Epidemiology Group), University of Aberdeen, Aberdeen, UK; 8 Department of Rheumatology and Clinical Immunology, Free University of Amsterdam, Amsterdam University Medical Centres, Amsterdam, The Netherlands; 9 Amsterdam UMC, Amsterdam Rheumatology and Immunology Center (ARC), Amsterdam, The Netherlands; 10 Reumaklinik Roskilde, Rheumatology Specialist Practice, Roskilde, Denmark; 11 ParHub RnD ApS, Copenhagen, Denmark; 12 Institute of Rheumatology, Prague, Czech Republic; 13 Department of Rheumatology, Charles University First Faculty of Medicine, Prague, Czech Republic; 14 Department of Internal Medicine—Nephrology, Rheumatology, Endocrinology, University Hospital Olomouc, Olomouc, Czech Republic; 15 Department of Medicine, University of Porto Faculty of Medicine, Porto, Portugal; 16 Rheumatology Department, Centro Hospitalar e Universitário de São João, Porto, Portugal; 17 Department of Rheumatology, ULSSM, Instituto Medicina Molecular, Faculdade de Medicina da Universidade de Lisboa, Centro Académico de Medicina de Lisboa, Hospital de Santa Maria, Lisbon, Portugal; 18 Department of Rheumatology, Hospital General, Universitario Gregorio Marañón, Madrid, Spain; 19 Faculty of Medicine, Complutense University of Madrid, Madrid, Spain; 20 Research Unit, Spanish Society of Rheumatology, Madrid, Spain; 21 Centre for Rheumatic Disease, University of Medicine and Pharmacy, Bucharest, Romania; 22 Department of Rheumatology, Division of Medicine, Turku University Hospital, Turku, Finland; 23 Department of Internal Medicine, University of Turku, Turku, Finland; 24 Rheumatology, Department of Medicine, Satakunta Central Hospital, Rauma, Finland; 25 Centre for Rheumatic Diseases, Tampere University Hospital, Tampere, Finland; 26 Faculty of Medicine and Health Technology, Tampere University, Tampere, Finland; 27 Department of Medicine, Kanta-Häme Central Hospital, Hämeenlinna, Finland; 28 Epidemiology, German Rheumatology Research Center, Berlin, Germany; 29 Department Rheumatology and Immunology, Inselspital, Bern University Hospital, University of Bern, Bern, Switzerland; 30 Department of Rheumatology, University Hospital Zurich, University of Zurich, Zürich, Switzerland; 31 Section for Rheumatology, Department for Neurology, Rheumatology and Physical Medicine, Helse Førde, Forde, Norway; 32 Public Health Section, University of Inland Norway, Elverum, Norway; 33 Department of Rheumatology, University Medical Centre Ljubljana, Ljubljana, Slovenia; 34 Faculty of Medicine, University of Ljubljana, Ljubljana, Slovenia; 35 Rheumatology Unit—Department of Precision and Regenerative Medicine and Ionian Area, University of Bari, Bari, Italy; 36 Rheumatology, Department of Clinical Internal, Anesthesiological and Cardiovascular Sciences, Sapienza, University of Rome, Rome, Italy; 37 Centre for Rheumatology Research, Landspítali—The National University Hospital of Iceland, Reykjavík, Iceland; 38 Faculty of Medicine, University of Iceland, Reykjavík, Iceland; 39 Clinical Epidemiology Division, Department of Medicine Solna, Karolinska Institute, Stockholm, Sweden; 40 Department of Rheumatology & Clinical Immunology and Department of Experimental Immunology, Amsterdam Institute for Immunology & Infectious Diseases, University of Amsterdam, Amsterdam, The Netherlands; 41 Amsterdam Rheumatology and Immunology Center, Reade and Amsterdam UMC, Amsterdam, The Netherlands; 42 Department of Internal Medicine, Division of Rheumatology, Dokuz Eylul University Faculty of Medicine, Izmir, Turkey

**Keywords:** Cardiovascular Disease, Hypertension, Psoriatic Arthritis, Biological Therapy, DMARD

## Abstract

**Objectives:**

To investigate associations between cardiometabolic comorbidities and clinical characteristics, prescription patterns and retention of first biologic/targeted synthetic disease-modifying anti-rheumatic drug (b/tsDMARD) in patients with psoriatic arthritis (PsA).

**Methods:**

Patients with PsA initiating a first b/tsDMARD treatment in 2015 or later were identified in eight European rheumatology registries. Patients with information on five cardiometabolic comorbidities (obesity, dyslipidaemia, diabetes, hypertension, ischaemic heart disease) at treatment start (baseline) were included. All analyses were conducted according to patients’ comorbidity burden (count: 0/1/≥2) and status (presence/absence of each comorbidity). Patient characteristics and prescription patterns were described. Twelve-month treatment retention rates were estimated and compared using Kaplan-Meier plots, log-rank tests and multivariable Cox regression analyses.

**Results:**

Among 5299 patients, 36% had at least one cardiometabolic comorbidity. Patients with comorbidity were older, had higher disease activity and more disability. Regardless of comorbidity, most patients were prescribed a tumour necrosis factor inhibitor (76%). The use of interleukin-17 inhibitors increased with comorbidity burden (0/1/≥2 comorbidities: 13%/18%/19%), whereas Janus kinase inhibitor use declined (2.3%/1.6%/0.8%). Retention rates were marginally lower with higher comorbidity burden (80%/76%/78%) (log-rank, p=0.036) and obesity (absent 79% vs present 77%) (log-rank, p=0.04). The risk of treatment withdrawal was only marginally higher in patients with higher comorbidity burden (one comorbidity: HR 1.19; 95% CI 1.02 to 1.40; ≥2 comorbidities: HR 1.18; 0.98 to 1.42).

**Conclusion:**

Patients with cardiometabolic comorbidities had higher disease activity at treatment initiation of the first b/tsDMARD. Prescription patterns varied with comorbidity burden. Cardiometabolic comorbidity burden, especially obesity, was associated with marginally lower treatment retention.

**WHAT IS ALREADY KNOWN ON THIS TOPIC**

- Psoriatic arthritis (PsA) is associated with cardiometabolic comorbidities, including obesity, dyslipidaemia, diabetes, hypertension and ischaemic heart disease.
- While obesity has been suggested to negatively impact certain biologic/targeted synthetic disease-modifying antirheumatic drug (b/tsDMARD) treatment outcomes, evidence remains limited on how the overall burden and the individual cardiometabolic comorbidities influence prescription patterns and treatment effectiveness in clinical practice.

**WHAT THIS STUDY ADDS**

- This real-world study in 5299 European patients with PsA is among the first to investigate associations between cardiometabolic comorbidities and clinical characteristics, prescription patterns and retention rate of first b/tsDMARD treatment.
- Patients with cardiometabolic comorbidities had higher disease activity and disability at treatment start.
- Differences in prescription patterns between patients with and without cardiometabolic comorbidities aligned well with current treatment recommendations.
- Comorbidity burden, especially obesity, was associated with marginally reduced treatment retention, but with small effect size warranting consideration of its clinical relevance.
- Dyslipidaemia, diabetes, hypertension and ischaemic heart disease were unassociated with retention.

**HOW THIS STUDY MIGHT AFFECT RESEARCH, PRACTICE OR POLICY**

- Cardiometabolic comorbidities challenge the pharmacological management of PsA.
- While choice of mode of action generally aligned with current treatment recommendations, the higher disease activity observed at treatment initiation may reflect delayed b/tsDMARD initiation or more severe inflammation in patients with cardiometabolic comorbidities.
- This suggests an unmet need in this patient group.

## Introduction

 Psoriatic arthritis (PsA) is a chronic, heterogeneous immune-mediated rheumatic disease characterised by inflammatory disease manifestations and a high comorbidity burden.^1^,^3^ The inflammation causes both musculoskeletal and non-musculoskeletal manifestations, including arthritis, spondylitis, enthesitis, dactylitis, skin and nail psoriasis, and may also affect the gut (inflammatory bowel disease) and the eyes (anterior uveitis).^2^ Compared with the general population, patients with PsA more often have cardiometabolic comorbidities, such as obesity, dyslipidaemia, diabetes, hypertension and ischaemic heart disease.^4^ Obesity, dyslipidaemia, diabetes and hypertension represent key components of metabolic syndrome,^5^ while ischaemic heart disease reflects established cardiovascular disease. Together, they frequently coexist with PsA and may share inflammatory pathways and genetic associations.^2 6 7^ In the PsA disease management, the associated cardiometabolic comorbidities require careful consideration.^8^,^10^

Over the past two decades, biological (b) and targeted synthetic (ts) disease-modifying antirheumatic drugs (DMARDs) with different modes of action and safety profiles have been approved for PsA treatment.^9 10^ Accordingly, pharmacological treatment recommendations have been developed by the European Alliance of Associations for Rheumatology (EULAR) and the Group for Research and Assessment of Psoriasis and Psoriatic Arthritis (GRAPPA).^9 10^ Importantly, these recommendations state that comorbidities should be taken into account when deciding on the treatment strategy, and it is further emphasised that more research is needed regarding the impact of comorbidities on drug choice.^9 10^ One concern is that cardiometabolic comorbidities, specifically obesity, could pose challenges in achieving the desired treatment outcomes.^9 10^ This has also been conceptualised in the recent EULAR definitions of difficult-to-manage PsA.^11^

While current evidence indicates that for certain treatments obesity is associated with impaired treatment outcomes in PsA,^12 13^ little is known regarding the other cardiometabolic comorbidities.^6^ In addition, observational studies have reported that an increased comorbidity burden negatively impacts treatment outcomes in PsA.^14^,^17^ Yet, the specific impact of cardiometabolic comorbidities on treatment effectiveness is unknown.

The European Spondyloarthritis Research Collaboration Network (EuroSpA) provides a unique opportunity to conduct real-world research on b/tsDMARD treatment effectiveness in PsA. Combining data from multiple registries ensures sufficient power for robust analyses. The collaboration currently includes European rheumatology registries that prospectively collect clinical data, including b/tsDMARD treatment outcomes and information on comorbidities, in patients with PsA followed in routine care.^18^,^20^

Thus, in European patients with PsA, who initiated first b/tsDMARD treatment, we aimed to investigate the association between five cardiometabolic comorbidities and (1) clinical patient characteristics, (2) the prescription patterns and (3) the 12-month retention rate of first b/tsDMARD. Comorbidities included obesity, dyslipidaemia, diabetes, hypertension and ischaemic heart disease, assessed as comorbidity burden and individually.

## Methods

This study was an observational cohort study conducted according to a predefined protocol.

### Data source

The study included secondary use of prospectively collected clinical data from rheumatology registries participating in the EuroSpA collaboration. Eight registries (Anti-TNF Therapy in Rheumatoid Arthritis (ATTRA), Czech Republic; Register of anti-rheumatic and Biological Therapy in Finland (ROBFIN), Finland; Rheumatoide Arthritis – Beobachtung der Biologika-Therapie (RABBIT-SpA), Germany; Norwegian Disease-Modifying Antirheumatic Drugs Register (NOR-DMARD), Norway; Reuma.pt, Portugal; Biorx.si, Slovenia; Spanish Registry for Adverse Events of Biological Therapy in Rheumatic Diseases (BIOBADASER), Spain; Swiss Clinical Quality Management in Rheumatic Diseases (SCQM), Switzerland) had available data on the presence (or absence) of the five cardiometabolic comorbidities (obesity, dyslipidaemia, diabetes, hypertension and ischaemic heart disease). Some registries used a list of predefined outcomes: Czech Republic (registry: ATTRA), Finland (ROBFIN, data available between 1 January 2019 and 31 December 2020), Germany (RABBIT-SpA), Slovenia (Biorx.si), Spain (BIOBADASER) and Switzerland (SCQM), or through Medical Dictionary for Regulatory Activities coding^21^: Portugal (Reuma.pt) and Spain (BIOBADASER). Finland (ROBFIN) used linked data until 31 December 2018, and Norway (NOR-DMARD) obtained data on comorbidities through linkage to other national registries. All data on comorbidities were collected at least since the initiation of the study period, except for data on dyslipidaemia from BIOBADASER, which were only collected starting in November 2022 as a predefined outcome. Details on registries and their recording practices of comorbidities have been reported elsewhere.^19 20 22^

The registries pseudonymised data before upload to a secure online server, where they were harmonised, quality-checked and pooled prior to statistical analyses.

### Case definition and exposure

We included patients with PsA, who initiated their first b/tsDMARD treatment from January 2015 to the data cut date in the individual registry (ranging from 12 August 2016 to 1 May 2024). We only included patients who were prescribed a b/tsDMARD approved for the treatment of PsA during the study period (table 1). Patients <18 years of age at the time of PsA diagnosis or initiation of the first b/tsDMARD were excluded. Patients were required to have data on the status (presence/absence) of the five comorbidities (obesity, dyslipidaemia, diabetes, hypertension and ischaemic heart disease) at the date of treatment start (=baseline). The definitions of comorbidities across the registries were largely similar (online supplemental table S1).

**Table 1:** Baseline characteristics of the overall cohort and according to comorbidity burden

	Overall N=5299		Comorbidity burden					
			No comorbidity N=3403		One comorbidity N=1055		Two or more comorbidities N=841	
	Results	Data availability	Results	Data availability	Results	Data availability	Results	Data availability
Age, years	56 (46, 64)	100%	53 (44, 61)	100%	58 (50, 65)	100%	63 (56, 70)	100%
Sex, female	2782 (53%)	100%	1756 (52%)	100%	574 (54%)	100%	452 (54%)	100%
BMI, kg/m^2^	27.6 (24.5, 31.5)	83%	26.0 (23.5, 28.7)	78%	30.1 (26.4, 33.2)	92%	32.3 (29.4, 36.2)	95%
Smoking status								
Never	2391 (54%)	83%	1511 (57%)	78%	487 (50%)	92%	393 (50%)	93%
Former	1106 (25%)	83%	594 (22%)	78%	268 (28%)	92%	244 (31%)	93%
Current	923 (21%)	83%	559 (21%)	78%	215 (22%)	92%	149 (19%)	93%
Years since diagnosis	9 (6, 14)	91%	9 (6, 14)	88%	9 (5, 14)	95%	10 (6, 16)	96%
Comorbidities								
Obesity	1005 (19%)	100%	0 (0%)	100%	449 (43%)	100%	556 (66%)	100%
Dyslipidaemia	503 (9.5%)	100%	0 (0%)	100%	106 (10%)	100%	397 (47%)	100%
Diabetes	349 (6.6%)	100%	0 (0%)	100%	70 (6.6%)	100%	279 (33%)	100%
Hypertension	1168 (22%)	100%	0 (0%)	100%	413 (39%)	100%	755 (90%)	100%
Ischaemic heart disease	121 (2.3%)	100%	0 (0%)	100%	17 (1.6%)	100%	104 (12%)	100%
Disease activity								
CRP, mg/L	5 (2, 13)	79%	5 (2, 11)	73%	6 (3, 15)	88%	8 (3, 17)	91%
28 SJC	3 (0, 6)	78%	2 (0, 5)	71%	4 (1, 8)	89%	4 (1, 8)	92%
28 TJC	4 (1, 10)	78%	3 (1, 8)	71%	6 (2, 11)	89%	8 (3, 12)	92%
DAPSA28	20 (13, 31)	41%	19 (12, 29)	43%	22 (15, 35)	43%	25 (17, 37)	33%
PGA, 0–10 mm	6 (4, 7)	51%	5 (3, 7)	55%	6 (5, 8)	47%	6 (4, 8)	37%
HAQ, 0–3	1.00 (0.50, 1.50)	70%	0.75 (0.38, 1.25)	62%	1.13 (0.63, 1.63)	83%	1.25 (0.88, 1.75)	88%
b/tsDMARD treatment start year								
2015–2017	1367 (26%)	100%	938 (28%)	100%	247 (23%)	100%	182 (22%)	100%
2018–2020	2012 (38%)	100%	1297 (38%)	100%	398 (38%)	100%	317 (38%)	100%
2021–2024	1920 (36%)	100%	1168 (34%)	100%	410 (39%)	100%	342 (41%)	100%

Data are presented as observed, median (25th–75th percentiles) unless otherwise stated. Comorbidity burden includes obesity, dyslipidaemia, diabetes, hypertension and ischaemic heart disease.

BMI, body mass index; b/tsDMARD, biologic/targeted synthetic disease-modifying antirheumatic drug; CRP, C reactive protein; DAPSA28, Disease Activity index for PSoriatic Arthritis in 28 joints; HAQ, Health Assessment Questionnaire; PGA, Patient Global Assessment; SJC, swollen joint count; TJC, tender joint count.

Patients were categorised based on (1) the comorbidity burden (no comorbidity, one comorbidity or two or more comorbidities) and (2) the status (presence/absence) of each individual comorbidity. A comorbidity was considered present if it had been recorded as present at any time point before or at baseline.

### Clinical baseline variables

Baseline data were collected from the clinical visit closest to baseline within a time window from up to 90 days before (preferred) to 30 days after the start date of the first b/tsDMARD treatment. The following clinical variables were extracted at baseline: age (years), sex (male/female), body mass index (BMI, kg/m^2^), smoking status (never; former; current), time since diagnosis (years), comorbidity status (presence/absence: obesity; dyslipidaemia; diabetes; hypertension; ischaemic heart disease, comorbidity definitions can be found in online supplemental table S1), C reactive protein (CRP, mg/L), Disease Activity index for PSoriatic Arthritis in 28 joints (DAPSA28),^23^ Patient Global Assessment (PGA, 0–10 on visual analogue scale), b/tsDMARD treatment (yes/no: tumour necrosis factor inhibitor (TNFi); interleukin (IL)-17i; IL-12/23i and IL-23i; Janus kinase inhibitor (JAKi); phosphodiesterase inhibitor (PDE4i)) and concomitant use of conventional synthetic DMARD (csDMARD) (yes/no: methotrexate; leflunomide; sulfasalazine). For each country/registry, we obtained data on the Purchasing Power Parity Gross Domestic Product (PPP GDP) per capita per €1000 in the year 2024 from the official website of the European Union.^24^

### Statistical analyses

Patients’ clinical characteristics and prescription patterns were assessed according to their comorbidity burden and the status of each individual comorbidity using clinical variables retrieved at baseline. The prescription patterns assessment was based on the drug class and compared numerically, which included bDMARDs (TNFi, IL-17i, IL-23/12i or IL-23i), tsDMARDs (JAKi, PDE4i) and concomitant csDMARDs. Descriptive statistics summarised baseline characteristics and prescription patterns, and a Venn diagram illustrated the overlap of comorbidities.

The 12-month follow-up period was defined as the time from baseline until treatment stop date, end of registry follow-up, death or date of last visit, whichever occurred first. Treatment retention rates stratified by baseline comorbidity burden and the status (presence/absence) of each individual comorbidity were estimated with Kaplan-Meier plots, and differences were assessed with log-rank test. The risk of treatment withdrawal at 12 months with increasing comorbidity burden was estimated and compared by multivariable Cox regression analysis with HRs. In the Cox model for comorbidity burden, we adjusted for baseline age, sex, calendar year and PPP GDP per capita to adjust for socioeconomic differences between registry countries. In a similar Cox regression model, we included the five individual comorbidities by substituting the comorbidity burden with the status (present/absent) of each comorbidity.

Observations were censored at the end of the 12-month follow-up period, and if patients withdrew due to remission and reasons other than adverse events or lack of effect, whichever came first. The proportional hazard assumption was tested for violations with Schoenfeld’s residuals.

Sensitivity analyses were performed by analysing the interaction effects of each comorbidity with age and sex, respectively. Further sensitivity analyses were performed by modifying the Cox models by (i) adding DAPSA28 as a covariate and (ii) removing the individual comorbidities as adjustment factors.

All analyses were performed using RStudio V.2022.02.2.

#### Missing values

All baseline covariates used in Cox models had complete information. In the sensitivity analyses, baseline DAPSA28 scores (missing data=59%) were imputed with multiple imputations with chained equations (MICE) and used as an additional covariate for the Cox models. For the imputation of DAPSA28, the individual components (28 tender joint counts (missing data=22%), 28 swollen joint counts (missing data=22%), CRP (missing data=21%), PGA (missing data=49%) and pain score (missing data=26%)) were imputed and subsequently used to compute the baseline DAPSA28 within the MICE model. The MICE model was set to use random predictive mean matching and create 40 datasets.

The study followed the Strengthening the Reporting of Observational Studies in Epidemiology (STROBE) guidelines.^25^

## Results

### Patient characteristics

Overall, 5299 patients with PsA and available data on the status for each of the five comorbidities were included. Patients who were excluded due to missing information on comorbidities (n=2064) generally had similar demographics compared with included patients, but tended to have lower disease activity and less available information on DAPSA28 and HAQ (online supplemental table S3). The proportion of patients that could be included varied between registries (online supplemental table S3).

Among the included patients, 36% had comorbidities, with 20% having one comorbidity and 16% having at least two comorbidities (table 1). Hypertension was the most frequent comorbidity (22%), followed by obesity (19%), dyslipidaemia (9.5%), diabetes (6.6%) and ischaemic heart disease (2.3%) (table 1).

**Figure 1:**
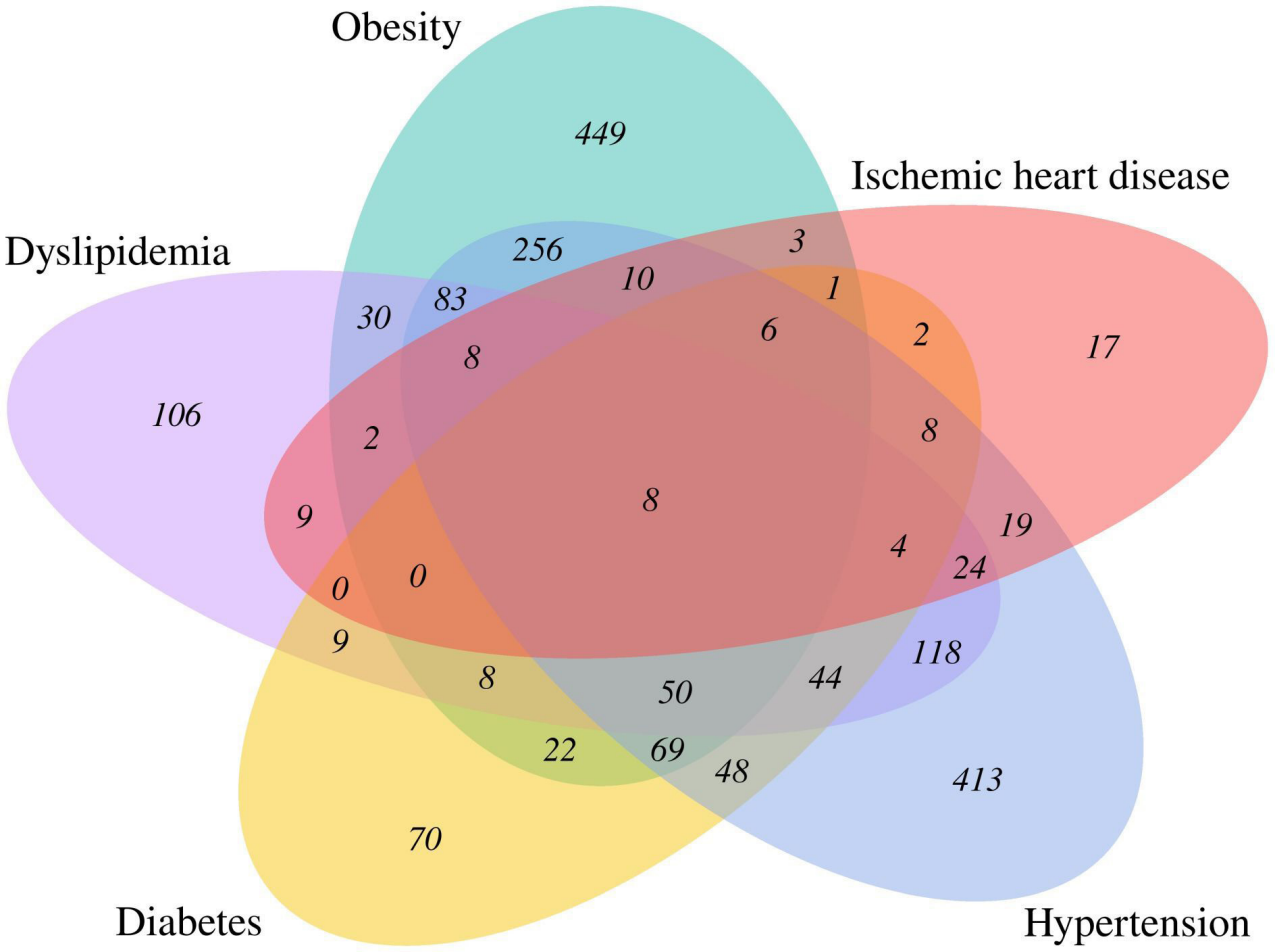
Venn diagram illustrating the overlap of comorbidities in 1896 patients with comorbidities. The sizes of the areas are arbitrary and do not reflect patient numbers.

Among patients with at least two comorbidities (n=841), a considerable overlap was found between having hypertension and being obese (58%, n=490), followed by having both hypertension and dyslipidaemia (40%, n=339) (figure 1, online supplemental table S4). Nearly half of the patients with obesity had no additional comorbidities (45%, n=449), whereas a majority of patients with ischaemic heart disease had at least one additional comorbidity (86%, n=104) (figure 1).

Compared with patients with no comorbidities, patients with higher comorbidity burden had numerically higher baseline age, higher disease activity (CRP, 28 swollen and tender joint counts, DAPSA28) and more functional disability (HAQ) (table 1). Similar results were found for the individual comorbidities, except for ischaemic heart disease, which was predominantly found in men, who had disease activity similar to those without ischaemic heart disease (online supplemental table S4).

### Prescription patterns

As shown in table 2, a bDMARD was prescribed in 93% of patients and a tsDMARD in 7%. Among the bDMARDs, TNFi was the most frequently prescribed drug class (76%), followed by IL-17i (15%). IL-12/23i and IL-23i were rarely prescribed (2.3%) (table 2). Patients with higher comorbidity burden were more likely to receive IL-17i as first bDMARD treatment (no comorbidity=13%, one comorbidity=18%, two or more comorbidities=19%). No major differences were observed in bDMARD prescription patterns across the individual comorbidities. Among the tsDMARDs, most patients received PDE4i (5.2%) and fewer JAKi (1.9%). PDE4i was prescribed largely independently of comorbidity burden (5.4%, 5.3%, 4.3%, respectively). However, it was prescribed less often in patients with dyslipidaemia (absent=5.5% vs present=2.6%), and more often in patients with ischaemic heart disease (5.1% vs 8.3%). JAKi was rarely prescribed in patients with higher comorbidity burden (2.3% vs 1.6% vs 0.8%, respectively) or with any of the individual comorbidities. Across individual registries, TNFi remained the most prescribed first b/tsDMARD. The increased use of IL-17i with higher comorbidity burden was not consistently observed across registries, and the use of PDE-4i varied between countries (online supplemental table S5).

**Table 2:** Prescription patterns of b/tsDMARD and concomitant csDMARD according to comorbidity burden and status of each individual comorbidity

Comorbidity		bDMARD, n (%)			tsDMARD, n (%)		b/tsDMARD with concomitant csDMARD, n (%)		
		TNFi	IL-17i	IL-12/23i or IL-23i	JAKi	PDE4i	MTX*	LEF*	SSZ*
Total number of patients, n=5299		4002 (76)	799 (15)	122 (2.3)	101 (1.9)	275 (5.2)	2646 (58)	556 (14)	474 (12)
No comorbidity, n=3.403		2617 (77)	452 (13)	74 (2.2)	77 (2.3)	183 (5.4)	1644 (59)	324 (14)	343 (15)
One comorbidity, n=1055		758 (72)	189 (18)	35 (3.3)	17 (1.6)	56 (5.3)	544 (55)	121 (13)	70 (7.7)
Two or more comorbidities, n=841		627 (75)	158 (19)	13 (1.5)	7 (0.8)	36 (4.3)	458 (57)	111 (15)	61 (8.0)
Obesity	Absent, n=4294	3282 (76)	588 (14)	101 (2.4)	93 (2.2)	230 (5.4)	2100 (58)	441 (15)	413 (15)
	Present, n=1005	720 (72)	211 (21)	21 (2.1)	8 (0.8)	45 (4.5)	546 (55)	115 (12)	61 (6.3)
Dyslipidaemia	Absent, n=4796	3603 (75)	722 (15)	109 (2.3)	100 (2.1)	262 (5.5)	2378 (58)	488 (14)	436 (12)
	Present, n=503	399 (79)	77 (15)	13 (2.6)	1 (0.2)	13 (2.6)	268 (58)	68 (16)	38 (9.0)
Diabetes	Absent, n=4950	3739 (76)	744 (15)	115 (2.3)	98 (2.0)	254 (5.1)	2446 (57)	523 (14)	448 (12)
	Present, n=349	263 (75)	55 (16)	7 (2.0)	3 (0.9)	21 (6.0)	200 (61)	33 (11)	26 (8.8)
Hypertension	Absent, n=4131	3148 (76)	581 (14)	98 (2.4)	82 (2.0)	222 (5.4)	2024 (58)	388 (13)	408 (14)
	Present, n=1168	854 (73)	218 (19)	24 (2.1)	19 (1.6)	53 (4.5)	622 (57)	86 (8.4)	148 (14)
Ischaemic heart disease	Absent, n=5178	3914 (76)	781 (15)	119 (2.3)	99 (1.9)	265 (5.1)	2587 (58)	538 (14)	461 (12)
	Present, n=121	88 (73)	18 (15)	3 (2.5)	2 (1.7)	10 (8.3)	59 (52)	18 (18)	13 (13)

Row percentages of number of patients are presented, where the distribution of b/tsDMARDs sums to 100%.

* The data availability for concomitant MTX is 87%, LEF is 76% and SSZ is 75%.

bDMARD, biologic disease-modifying antirheumatic drug; csDMARD, conventional synthetic disease-modifying antirheumatic drug; IL, interleukin; JAKi, Janus kinase inhibitor; LEF, leflunomide; MTX, methotrexate; PDE4i, phosphodiesterase 4 inhibitor; SSZ, sulfasalazine; TNFi, tumour necrosis factor inhibitor; tsDMARD, targeted synthetic disease-modifying antirheumatic drug.

Overall, more than half of the patients received concomitant methotrexate, regardless of comorbidity burden or status (table 2). However, methotrexate was less frequently prescribed in patients with ischaemic heart disease (58% vs 52%), leflunomide less frequently in patients with hypertension (13% vs 8.4%), while sulfasalazine was less commonly used in those with higher comorbidity burden (15% vs 7.7% vs 8.0%), obesity (15% vs 8.8%), dyslipidaemia (12% vs 9.0%) or diabetes (12% vs 8.8%).

### Treatment retention

**Figure 2:**
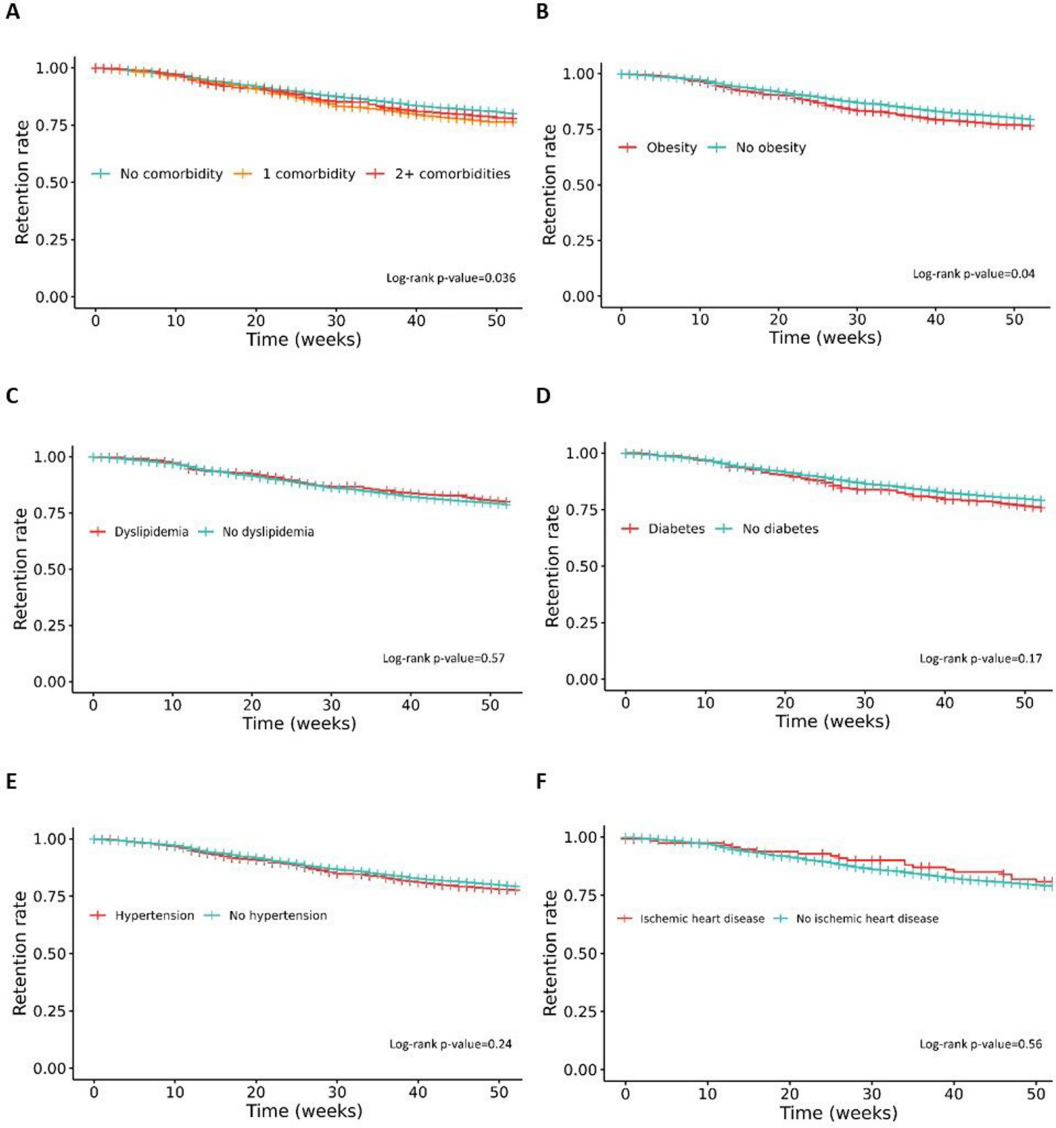
Twelve-month retention rates with Kaplan-Meier plots and log-rank test. Patients with psoriatic arthritis categorised by (A) comorbidity burden, (**B**) obesity, (**C**) dyslipidaemia, (**D**) diabetes, (**E**) hypertension and (F) ischaemic heart disease.

The retention rates of the first b/tsDMARD treatment at 12 months were 80% (95% CI 79% to 81%) in patients with no comorbidity, 76% (74% to 79%) with one comorbidity and 78% (75% to 81%) with two or more comorbidities (figure 2A). The retention rates were lower in patients with one comorbidity or two or more comorbidities versus no comorbidities (log-rank, p=0.036). For the individual comorbidities, retention rates were: in presence of obesity 77% (74% to 80%) versus in absence of obesity 79% (78% to 81%), for dyslipidaemia 80% (76% to 84%) versus 79% (78% to 80%), for diabetes 76% (71% to 81%) versus 79% (78% to 80%), for hypertension 78% (75% to 80%) vs 79% (78% to 81%) and for ischaemic heart disease 81% (74% to 89%) vs 79% (78% to 80%), respectively (figure 2B–F). Retention was lower in patients with versus without obesity (log-rank, p=0.04), while no differences were found for the other comorbidities.

**Figure 3:**
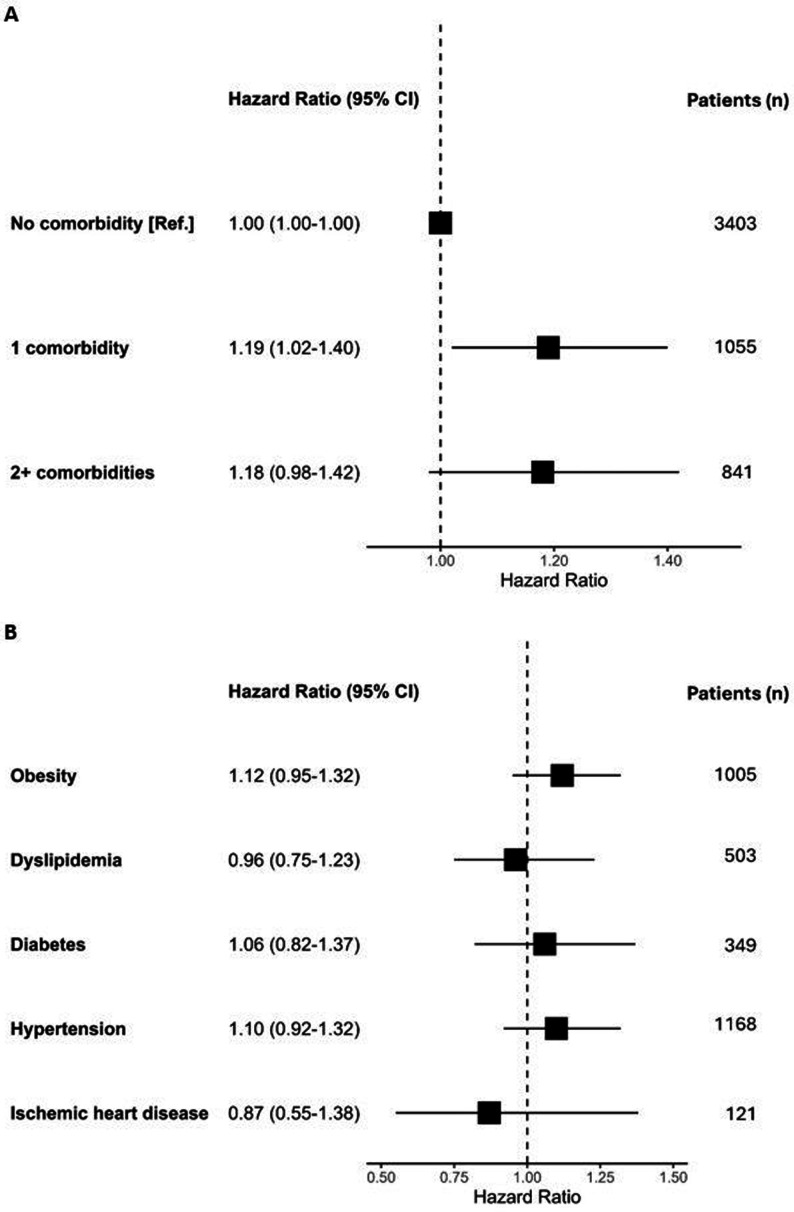
First biologic/targeted synthetic disease-modifying antirheumatic drug treatment withdrawal during 12-month follow-up by (A) comorbidity burden and (B) individual comorbidities. Forest plot of multivariable Cox regression analysis with HRs (95% CI) adjusted for baseline age, sex, calendar year of treatment start and purchasing power-adjusted gross domestic product per capita per €1000.

In the Cox regression analysis, the risk of withdrawal from treatment during 12-month follow-up was significantly higher for patients with one comorbidity (HR 1.19; 95% CI 1.02 to 1.40) and numerically higher for those with two or more comorbidities (HR 1.18; 95% CI 0.98 to 1.42), as compared with no comorbidity (figure 3). In the model including the status of the five individual comorbidities, no differences were found in the HR for treatment withdrawal (figure 3).

In the sensitivity analyses, no significant interactions or associations in the Cox regression analyses were observed (data not shown). The proportional hazard assumptions were violated for the sex covariate. However, stratifying by sex revealed no significant differences, as the HR estimates remained close to 1. Furthermore, the HR estimates did not change when omitting the individual comorbidities or when including DAPSA28 as a covariate.

## Discussion

In this large European cohort study of >5000 European real-world patients with PsA, we were able to investigate the association between five cardiometabolic comorbidities and patient characteristics, prescription patterns and retention of the first b/tsDMARD treatment. Almost one-third of patients had at least one cardiometabolic comorbidity, of which hypertension and obesity were the most frequent.

A key finding of our study was that a high cardiometabolic comorbidity burden was associated with higher disease activity and more disability at the time of b/tsDMARD treatment initiation. This is in line with previous studies demonstrating an association between comorbidities in general (ie, including non-cardiometabolic comorbidities) and PsA disease activity,^1415 26^,^28^ including one study with around 1500 patients particularly having cardiovascular comorbidities or metabolic syndrome (obesity, dyslipidaemia, diabetes, hypertension).^28^ The finding may reflect a higher threshold for initiating b/tsDMARD treatment in patients with cardiometabolic comorbidities,^29^ resulting in more severe disease at treatment start. This is notable given the EULAR recommendations on cardiovascular risk management advise optimal control of PsA disease activity to reduce cardiovascular risk.^8^ These recommendations are supported by studies suggesting that reducing inflammation and achieving PsA disease control with TNFi or other bDMARDs may also reduce the risk of cardiovascular events.^30^,^32^ Furthermore, our finding suggests that inflammation from the musculoskeletal manifestations of PsA together with the low-grade inflammation from the cardiometabolic diseases may interact synergistically and contribute to a higher disease activity.^27 33 34^

When it comes to prescription patterns, our findings show that clinical practice is largely in agreement with the latest EULAR treatment recommendations,^10^ which prioritise TNFi as the first option, followed by IL-17i, IL-23i or IL-12/23i, JAKi and PDE4i, depending on specific PsA disease manifestations, risk profiles and comorbidities. However, while some antirheumatic treatments, such as corticosteroids and non-steroidal anti-inflammatory drugs, are known to increase cardiovascular risk despite their anti-inflammatory abilities, concerns have also been raised regarding DMARDs with newer modes of action.^35 36^ Regardless of the comorbidity burden and status, TNFi was the preferred mode of action, and it was prescribed to more than seven out of 10 patients. Compared with patients without comorbidities, more patients with comorbidities received IL-17i, which may be partly attributed to the known association between severe psoriasis and an elevated risk of CV disease.^37^ Current EULAR and GRAPPA treatment guidelines recommend IL-17i for patients with clinically relevant skin involvement.^9 10^ However, we did not have data to explore this further. In addition, TNFi are used with caution in patients with moderate to severe heart failure, which may further influence treatment selection in patients with a high cardiometabolic comorbidity burden.^9 38^ Although heart failure was not assessed in the present study, such considerations in routine care may contribute to the increased use of IL-17i in this patient group. We did not find a specific pattern regarding IL-23i and IL-12/23i, other than overall few patients received those drugs as first b/tsDMARD treatment. JAKis were rarely prescribed and even less so in patients with cardiometabolic comorbidities. This is in accordance with concerns raised following results from the ORAL surveillance study, which reported increased risk of cardiovascular events in older patients with rheumatoid arthritis who had cardiovascular risk factors.^9 10 36^ The GRAPPA recommendations suggest no hierarchy between the b/tsDMARDs, but recommend caution with using JAKi among patients with elevated cardiovascular risk.^9^ However, the limited use may also be explained by our study period, which began in January 2015, whereas the first JAKi was approved by the European Medicines Agency for PsA in June 2018. PDE4i was prescribed in a relatively high proportion of patients, especially among those with ischaemic heart disease. This probably reflects that, despite its lower efficacy compared with other bDMARDs, PDE4i has advantages such as oral administration and a favourable safety profile with minimal need for monitoring.^39^ Across individual registries, TNFi remained the most prescribed first b/tsDMARD. However, the increased use of IL-17i with higher comorbidity burden was not consistently observed across registries. For example, the use of IL-17i was overall low in Norway and Portugal, where IL-17i is not specifically prioritised based on skin involvement in the national recommendations.^40^ In contrast, the relatively higher use of PDE-4i in Switzerland was likely to reflect the approval of PDE-4i at a similar treatment line as other b/tsDMARDs.^40^ These observations suggest that for the individual registry, national treatment recommendations, reimbursement policies and registry composition case mix influence prescription patterns, which may therefore deviate from the overall finding.

The presence of one or several cardiometabolic comorbidities at b/tsDMARD treatment start was associated with marginally poorer treatment retention, compared with patients without such comorbidities. This finding appeared to be driven mainly by obesity, as patients with obesity experienced a poorer drug retention rate than patients without obesity, and nearly half of the patients with obesity had no additional comorbidity. Although these findings were statistically significant, the small effect size warrants consideration of its clinical relevance. No uniform method exists for measuring a cardiometabolic comorbidity burden that takes the severity of individual comorbidities into account. We used counts of five specific comorbidities, whereas other studies applied selected comorbidity indices developed for various research contexts. For example, a real-world study of 208 patients with PsA reported lower b/tsDMARD retention rate and higher risk of treatment withdrawal in patients with higher comorbidity burden, assessed with the modified Rheumatic Disease Comorbidity Index (RDCI).^16^ This index includes obesity, diabetes, hypertension, myocardial infarction and other cardiovascular and chronic diseases.^41^ The findings are in line with other real-world single-centre studies examining the impact of comorbidity burden, defined by counts or the Charlson Comorbidity Index (CCI), on first TNFi treatment outcomes.^15 17 42 43^ A few smaller studies in PsA, each with fewer than 200 patients, found similar results across a range of comorbidity indices (CCI, RDCI, counts) and b/tsDMARD treatments.^14 16^ Studies on IL-17i reported either no difference or even improved treatment effectiveness in patients with comorbidities.^26 44 45^ However, the results of these studies should be interpreted with caution due to their low patient numbers.

Several mechanisms may underlie the observed association between cardiometabolic comorbidities and poorer treatment retention. Obesity, in particular, has been linked to altered pharmacokinetics of bDMARDs (including TNFi, IL-23i and IL-17Ai) in patients with rheumatic diseases, including increased volume of distribution and lower circulating drug concentrations, which may reduce treatment effectiveness.^7 12^ In addition, adipose tissue is an active immunological organ that contributes to a pro-inflammatory milieu through the secretion of adipokines and cytokines, potentially counteracting therapeutic effects of b/tsDMARDs.^7^ Diabetes has also been associated with abnormal pro-inflammatory cytokine production,^46^ which may contribute to loss of response and treatment discontinuation of b/tsDMARDs. Beyond biological mechanisms, cardiometabolic comorbidities may influence treatment retention through an increased risk of adverse events, polypharmacy and physician or patient preferences leading to earlier discontinuation. These mechanisms could not be directly assessed in the present study and warrant further investigation.

Our study did not show any clinically relevant increased risk of treatment withdrawal in patients with PsA and hypertension, dyslipidaemia, diabetes or ischaemic heart disease. In other PsA studies, obesity has been suggested to impair the treatment outcomes of TNFi,^12 13 43^ while contradictory results have been reported for IL-17i.^4445 47^,^50^ Two studies found that hypertension reduced the TNFi treatment response,^14 43^ whereas little is known regarding other cardiometabolic comorbidities. Future studies are needed to further investigate the association between cardiometabolic comorbidities and treatment outcomes, and to clarify whether obesity alone is the primary driver of impaired treatment response or whether the overall cardiometabolic comorbidity burden plays a larger role.

This study had several strengths. It leveraged data pooled from multiple European rheumatology registries, providing a large sample of real-world patients with PsA. Additionally, we had access to PsA disease activity measures as well as other important clinical data. The study also had limitations. The study relied partially on registration of comorbidities by physicians, which may have introduced bias. Thus, misclassification could result from patient recall bias or incomplete data registration by the rheumatologists (ie, typically patients with comorbidities registered as not having them), which is a well-known limitation in studies based on data collected in routine care. This would contribute to an underestimation of the prevalence of certain cardiometabolic comorbidities in our study. Moreover, selection bias may have affected our cohort, as rheumatologists might have been less inclined to report comorbidity status in patients without comorbidities, leading to patient exclusion from our study. For the Spanish BIOBADASER registry, data on dyslipidaemia were only recently collected, beginning in November 2022. Consequently, we had to exclude some Spanish patients due to missing data on dyslipidaemia. Compared with our findings, a previous systematic review and meta-analysis reported higher prevalences of cardiometabolic comorbidities among patients with PsA, including obesity (27% vs 19%), dyslipidaemia (28% vs 9.5%), diabetes (11% vs 6.6%), hypertension (29% vs 22%) and ischaemic heart disease (7% vs 2.3%).^3^ Besides recall bias, the inclusion criteria and variation in comorbidity data sources across registries may have led to an underestimation of cardiometabolic comorbidity prevalence in our study. Differences may arise from which time point the information was collected. In our study, it was collected before initiation of the first b/tsDMARD, in other studies this may have been later in the disease course, where the comorbidity profile may have changed (worsened). Furthermore, other relevant cardiovascular risk factors, diseases and metabolic comorbidities (eg, hyperuricaemia, metabolic associated fatty liver disease, cerebrovascular disease, arrhythmias, peripheral arterial disease, etc) were not included because few or no registries collected those data. This may have led to an underestimation of the overall cardiometabolic burden in the study population.

In conclusion, European real-world patients with PsA and cardiometabolic comorbidities had higher disease activity and increased disability at b/tsDMARD treatment start, which may reflect delayed b/tsDMARD initiation or more severe inflammation in patients with cardiometabolic comorbidities. Prescription patterns of first b/tsDMARD were largely in line with current treatment recommendations. Comorbidity burden, especially obesity, was associated with marginally lower treatment retention.

## Supplementary material

10.1136/rmdopen-2025-006477 online supplemental file 1

## Data Availability

All data relevant to the study are included in the article or uploaded as supplementary information.
