# Supplementary material for: Impact of cardiometabolic comorbidities on clinical characteristics, prescription patterns and retention rate of first b/tsDMARD treatment in 5299 European real-world patients with psoriatic arthritis
Source: RMD Open. 2026 May 7;12(2):e006477. doi: 10.1136/rmdopen-2025-006477 (PMC13157805; doi:10.1136/rmdopen-2025-006477)
Supplement: online supplemental file 1 [file rmdopen-12-2-s001.docx]

**Supplementary material**

**Supplementary Table S1. Identification of comorbidities from pre-defined outcome lists, ICD10-codes, MedDRA-codes, or other methods used by the individual rheumatology registries**

|  | **Pre-defined outcome list** | **ICD-10** | **MedDRA** | **Other** |
| --- | --- | --- | --- | --- |
| **Registries** | See below parentheses | RobFin; NOR-DMARD | Reuma.pt; BIOBADASER | See below parentheses |
| **Obesity** |  | E66 | 10029883  10059610 | Calculated BMI ≥30 kg/m^2^ (ATTRA; Biorx.si; NOR-DMARD; RABBIT-SpA; Reuma.pt; RobFIN; BIOBADASER; SCQM) |
| **Dyslipidemia** | Hyperlipidemia (ATTRA; RABBIT-SpA, Rob-FIN; BIOBADASER; SCQM)  Hypercholesterolemia (Rob-FIN)  Dyslipidemia (Rob-FIN) | E78 | 10058108 | Deduced from medication (Biorx.si) |
| **Diabetes*** | Diabetes mellitus (ATTRA; Biorx.si; Rob-FIN; BIOBADASER)  Type 1 Diabetes mellitus (RABBIT-SpA; Rob-FIN; SCQM)  Type 2 Diabetes mellitus (RABBIT-SpA; Rob-FIN; SCQM) | E10-E14 | 10012601  10012607  10012608  10012613  10022491  10049746  10053247  10067584 10067585 10072628 10081755 | Deduced from medication (Biorx.si) |
| **Hypertension** | Hypertension (ATTRA; Biorx.si, RABBIT-SpA; Rob-FIN; BIOBADASER; SCQM) | I10-I13, I15 | 10000358  10012758  10015488  10020772  10038464  10038562  10039834  10042957  10049079 | Deduced from medication (Biorx.si) |
| **Ischemic heart disease**** | Coronary artery disease (ATTRA)  Coronary heart disease (RABBIT-SpA; SCQM)  Myocardial infarction (ATTRA; Biorx.si; Rob-FIN; BIOBADASER; SCQM)  Angina pectoris (Biortx.si; Rob-FIN)  Ischemic heart disease (Biorx.si; Rob-FIN; BIOBADASER) | I20- I25 | 10000891  10002383  10002388  10003211  10011077  10011078  10011086  10011089  10028596  10028600  10048858  10050329  10051592  10052086  10052895  10058144  10065420  10077824 |  |
| *Diabetes registration differed across registries and was defined based on information on Diabetes Mellitus Type 1, Diabetes Mellitus Type 2, and Diabetes undifferentiated. **Ischemic heart disease registration differed across registries and was defined based on information on angina pectoris, myocardial infarction, and ischemic heart disease undifferentiated. Abbreviations: **BMI** body mass index; **ICD** International Classification of Diseases; **MedDRA** Medical Dictionary for Regulatory Activities. | | | | |

**Supplementary Table S2. Ethical approvals of participating registries.**

| **Registry** | **Country** | **Ethical approval** | **Reference** |
| --- | --- | --- | --- |
| ATTRA | Czech Republic | Ethical approval for ATTRA was granted by the Czech Multicentre Research Ethics Committee. No additional ethical approval was required for the current analysis. All subjects provided their written consent for the collection and storage of data before participation. | No. 201611 S300. |
| BIOBADASER | Spain | Ethical approval for BIOBADASER was initially granted by the Research Ethics Committee of the Hospital Clínic de Barcelona, while the Research Ethics Committee of the Hospital de Canarias is the current Reference Committee. No additional ethical approval was required for the current analysis. Written informed consent was obtained from all patients. | FER-ADA-2015-01 |
| biorx.si | Slovenia | Obtained from National Ethics Committee at Ministry of Health. | Approval No.: 0120-191/2016/2 and 0120-118/2023/6 |
| NOR-DMARD | Norway | Ethical approval has been granted for the registry and all patients have provided informed consent. | REK 2011/1339 and 105-00055 |
| RABBIT-SpA | Germany | RABBIT-SpA received approval by the Ethics Committee of the Charité University Medicine, Berlin | #EA1/246/16 |
| Reuma.pt | Portugal | Ethical approval has been granted for the registry and all patients provide informed consent and authorize the use of their personal data for investigation, which must be approved by an ethics committee (no local approval required). | Not applicable |
| ROBFIN | Finland | Ethical approval granted by the coordinating ethical committee of Helsinki and Uusimaa Hospital District | 73/13/03/00/14 |
| SCQM | Switzerland | Ethical approval has been granted by the Ethics Committee of the Canton of Zurich.  Written informed consent was obtained from all patients. | BASEC 2022-00272 |

**Supplementary Table S3. Baseline characteristics of included and excluded patients**

|  | Included patients  N=5,299 | | Excluded patients  N=2,064 | |
| --- | --- | --- | --- | --- |
|  | Results | Availability | Results | Availability |
| ATTRA (Czech Republic) | 1,418 (27%) |  | 62 (3%) |  |
| BIOBADASER (Spain) | 109 (2%) |  | 1,340 (65%) |  |
| BIORX.SI (Slovenia) | 360 (7%) |  | 0 (0%) |  |
| NOR-DMARD (Norway) | 237 (4%) |  | 296 (14%) |  |
| RABBIT-SpA (Germany) | 830 (16%) |  | 8 (1%) |  |
| Reuma.pt (Portugal) | 1,207 (23%) |  | 0 (0%) |  |
| ROBFIN (Finland) | 806 (15%) |  | 0 (0%) |  |
| SCQM (Switzerland) | 332 (6%) |  | 358 (17%) |  |
| Age, years | 56 (46, 64) | 100% | 56 (47, 64) | 100% |
| Sex, female | 2,782 (53%) | 100% | 1,064 (52%) | 100% |
| BMI, kg/m^2^ | 27.6 (24.5, 31.5) | 83% | 27.8 (24.7, 31.7) | 79% |
| Smoking status |  |  |  |  |
| Never | 2,391 (54%) | 83% | 463 (26%) | 87% |
| Former | 1,106 (25%) |  | 959 (54%) |  |
| Current | 923 (21%) |  | 367 (21%) |  |
| Years since diagnosis | 9 (6, 14) | 91% | 8 (5, 12) | 93% |
| *Comorbidities* | | | | |
| Obesity | 1,005 (19%) | 100% | 433 (29%) | 73% |
| Dyslipidemia | 503 (9.5%) | 100% | 43 (15%) | 14% |
| Diabetes | 349 (6.6%) | 100% | 103 (6.6%) | 76% |
| Hypertension | 1,168 (22%) | 100% | 303 (18%) | 81% |
| Ischemic heart disease | 121 (2.3%) | 100% | 40 (2.6%) | 75% |
| *Disease activity* | | | | |
| CRP, mg/L | 5 (2, 13) | 79% | 3 (0, 8) | 83% |
| 28 swollen joint count | 3 (0, 6) | 78% | 2 (0, 4) | 82% |
| 28 tender joint count | 4 (1, 10) | 78% | 3 (1, 6) | 82% |
| DAPSA28 | 20 (13, 31) | 41% | 16 (10, 24) | 17% |
| PGA, 0-10 mm | 6 (4, 7) | 51% | 6 (4, 7) | 75% |
| HAQ, 0-3 | 1.00 (0.50, 1.50) | 70% | 0.63 (0.13, 1.00) | 24% |
| *Treatment* | | | | |
| b/tsDMARD treatment start year |  |  |  |  |
| 2015-2017 | 1,367 (26%) | 100% | 522 (25%) | 100% |
| 2018-2020 | 2,012 (38%) | 100% | 759 (37%) | 100% |
| 2021-2024 | 1,920 (36%) | 100% | 783 (38%) | 100% |
| TNFi | 4,002 (76%) | 100% | 1,537 (74%) | 100% |
| IL-17i | 799 (15%) | 100% | 226 (11%) | 100% |
| IL-12/23i or IL-23i | 122 (2.3%) | 100% | 76 (3.7%) | 100% |
| JAKi | 101 (1.9%) | 100% | 29 (1.4%) | 100% |
| PDE4i | 275 (5.2%) | 100% | 196 (9.5%) | 100% |
| Concomitant csDMARD |  |  |  | 100% |
| MTX | 2,646 (58%) | 87% | 907 (47%) | 93% |
| LEF | 556 (14%) | 76% | 273 (15%) | 86% |
| SSZ | 474 (12%) | 75% | 118 (6.6%) | 86% |
| Data are as observed, median (25%, 75% percentiles) unless otherwise stated. Abbreviations: bDMARD, biologic disease-modifying anti-rheumatic drug; BMI, Body Mass Index; CRP, C-reactive protein; csDMARD, conventional synthetic disease-modifying anti-rheumatic drug; DAPSA28, Disease Activity index for PSoriatic Arthritis in 28 joints; HAQ, Health Assessment Questionnaire; i, inhibitor; IL, interleukin; JAKi, Januskinase inhibitor; LEF, leflunomide; MTX, methotrexate; PDE4i, Phosphodiesterase 4 inhibitor; PGA, Patient Global Assessment; TNFi, Tumor Necrosis Factor inhibitor; tsDMARD, targeted synthetic disease-modifying anti-rheumatic drug; SSZ, Sulfasalazine. | | | | |

**Supplementary Table S4.** **Baseline characteristics of patients according to the presence or absence of each cardiometabolic comorbidity**

|  | Obesity | | | | Dyslipidemia | | | |
| --- | --- | --- | --- | --- | --- | --- | --- | --- |
|  | **Absent (N=4,294)** | | **Present (N=1,005)** | | **Absent (N=4,796)** | | **Present (N=503)** | |
|  | **Results** | **Availability** | **Results** | **Availability** | **Results** | **Availability** | **Results** | **Availability** |
| Age, years | 55 (46, 63) | 100% | 57 (50, 64) | 100% | 55 (46, 63) | 100% | 63 (57, 70) | 100% |
| Sex, female | 2,203 (51%) | 100% | 579 (58%) | 100% | 2,527 (53%) | 100% | 255 (51%) | 100% |
| BMI, kg/m^2^ | 26.2 (23.8, 28.7) | 80% | 33.6 (31.5, 37.2) | 98% | 27.4 (24.4, 31.2) | 83% | 29.3 (25.7, 33.2) | 92% |
| Smoking status |  |  |  |  |  |  |  |  |
| Never | 1,954 (56%) | 80% | 437 (46%) | 94% | 2,150 (54%) | 82% | 241 (51%) | 94% |
| Former | 824 (24%) |  | 282 (30%) |  | 958 (24%) |  | 148 (31%) |  |
| Current | 695 (20%) |  | 228 (24%) |  | 839 (21%) |  | 84 (18%) |  |
| Years since diagnosis | 9 (6, 14) | 89% | 9 (5, 14) | 97% | 9 (6, 14) | 90% | 10 (6, 17) | 97% |
| *Comorbidities* | | | | | | | | |
| Obesity | 0 (%) | 100% | 1,005 (100%) | 100% | 816 (17%) | 100% | 189 (38%) | 100% |
| Dyslipidemia | 314 (7.3%) | 100% | 189 (19%) | 100% | 0 (0%) | 100% | 503 (100%) | 100% |
| Diabetes | 185 (4.3%) | 100% | 164 (16%) | 100% | 226 (4.7%) | 100% | 123 (24%) | 100% |
| Hypertension | 678 (16%) | 100% | 490 (49%) | 100% | 829 (17%) | 100% | 339 (67%) | 100% |
| Ischemic heart disease | 83 (1.9%) | 100% | 38 (3.8%) | 100% | 66 (1.4%) | 100% | 55 (11%) | 100% |
| *Disease activity* | | | | | | | | |
| CRP, mg/L | 5 (2, 12) | 75% | 9 (4, 17) | 93% | 5 (2, 13) | 78% | 7 (3, 16) | 87% |
| 28 SJC | 2 (0, 6) | 74% | 4 (1, 8) | 95% | 2 (0, 6) | 77% | 5 (1, 8) | 89% |
| 28 TJC | 4 (1, 8) | 74% | 7 (3, 12) | 95% | 4 (1, 9) | 77% | 8 (3, 13) | 89% |
| DAPSA28 | 20 (13, 30) | 43% | 24 (16, 36) | 35% | 20 (13, 31) | 43% | 25 (14, 41) | 25% |
| PGA, 0-10 mm | 6 (3, 7) | 53% | 6 (5, 8) | 40% | 6 (4, 7) | 53% | 6 (4, 8) | 29% |
| HAQ, 0-3 | 0.88 (0.38, 1.38) | 65% | 1.25 (0.88, 1.75) | 91% | 1.00 (0.38, 1.50) | 69% | 1.25 (0.88, 1.75) | 83% |
| *b/tsDMARD treatment start year* | | | | | | | | |
| 2015-2017 | 1,178 (27%) | 100% | 189 (19%) | 100% | 1,252 (26%) | 100% | 115 (23%) | 100% |
| 2018-2020 | 1,616 (38%) | 100% | 396 (39%) | 100% | 1,845 (38%) | 100% | 167 (33%) | 100% |
| 2021-2024 | 1,500 (35%) | 100% | 420 (42%) | 100% | 1,699 (35%) | 100% | 221 (44%) | 100% |
| Data are as observed, median (25%, 75% percentiles) unless otherwise stated. Abbreviations: BMI, Body Mass Index; CRP, C-reactive protein; DAPSA28, Disease Activity index for PSoriatic Arthritis in 28 joints; HAQ, Health Assessment Questionnaire; PGA, Patient Global Assessment; SJC swollen joint count; TJC tender joint count. | | | | | | | | |

**Supplementary Table S4 (continued). Baseline characteristics of patients according to the presence or absence of each cardiometabolic comorbidity**

|  | Diabetes | | | | Hypertension | | | |
| --- | --- | --- | --- | --- | --- | --- | --- | --- |
|  | **Absent (N=4,950)** | | **Present (N=349)** | | **Absent (N=4,131)** | | **Present (N=1,168)** | |
|  | **Results** | **Availability** | **Results** | **Availability** | **Results** | **Availability** | **Results** | **Availability** |
| Age, years | 55 (46, 63) | 100% | 64 (57, 71) | 100% | 53 (44, 61) | 100% | 63 (56, 70) | 100% |
| Sex, female | 2,595 (52%) | 100% | 187 (54%) | 100% | 2,153 (52%) | 100% | 629 (54%) | 100% |
| BMI, kg/m^2^ | 27.4 (24.4, 31.1) | 83% | 31.2 (27.5, 35.5) | 92% | 26.9 (24.1, 30.4) | 81% | 30.1 (26.5, 34.1) | 93% |
| Smoking status |  |  |  |  |  |  |  |  |
| Never | 2,225 (54%) | 83% | 166 (52%) | 92% | 1,826 (55%) | 81% | 565 (52%) | 92% |
| Former | 1,006 (25%) |  | 100 (31%) |  | 783 (23%) |  | 323 (30%) |  |
| Current | 869 (21%) |  | 54 (17%) |  | 734 (22%) |  | 189 (18%) |  |
| Years since diagnosis | 9 (6, 14) | 90% | 9 (6, 16) | 95% | 9 (6, 13) | 89% | 10 (6, 17) | 78% |
| *Comorbidities* | | | | | | | | |
| Obesity | 841 (17%) | 100% | 164 (47%) | 100% | 515 (12%) | 100% | 490 (42%) | 100% |
| Dyslipidemia | 380 (7.7%) | 100% | 123 (35%) | 100% | 164 (4.0%) | 100% | 339 (29%) | 100% |
| Diabetes |  | 100% |  | 100% | 112 (2.7%) | 100% | 237 (20%) | 100% |
| Hypertension | 931 (19%) | 100% | 237 (68%) | 100% | 0 (%) | 100% | 1,168 (100%) | 100% |
| Ischemic heart disease | 92 (1.9%) | 100% | 29 (8.3%) | 100% | 34 (0.8%) | 100% | 87 (7.4%) | 100% |
| *Disease activity* | | | | | | | | |
| CRP, mg/L | 5 (2, 13) | 78% | 8 (3, 16) | 87% | 5 (2, 12) | 76% | 8 (3, 16) | 89% |
| 28 SJC | 3 (0, 6) | 78% | 4 (1, 8) | 87% | 2 (0, 6) | 75% | 4 (1, 8) | 90% |
| 28 TJC | 4 (1, 9) | 78% | 6 (2, 12) | 87% | 4 (1, 8) | 75% | 7 (3, 12) | 90% |
| DAPSA28 | 20 (13, 31) | 42% | 24 (18, 39) | 40% | 19 (13, 30) | 43% | 25 (17, 36 | 37% |
| PGA, 0-10 mm | 6 (4, 7) | 51% | 6 (4, 8) | 44% | 6 (3, 7) | 53% | 6 (4, 8) | 42% |
| HAQ, 0-3 | 1.00 (0.50, 1.50) | 70% | 1.38 (0.88, 1.75) | 81% | 0.88 (0.38, 1.38) | 66% | 1.25 (0.75, 1.75) | 85% |
| *b/tsDMARD treatment start year* | | | | | | | | |
| 2015-2017 | 1,277 (26%) | 100% | 90 (26%) | 100% | 1,101 (27%) | 100% | 266 (23%) | 100% |
| 2018-2020 | 1,889 (38%) | 100% | 123 (35%) | 100% | 1,562 (38%) | 100% | 450 (39%) | 100% |
| 2021-2024 | 1,784 (36%) | 100% | 136 (39%) | 100% | 1,468 (36%) | 100% | 452 (39%) | 100% |
| Data are as observed, median (25%, 75% percentiles) unless otherwise stated. Abbreviations: BMI, Body Mass Index; CRP, C-reactive protein; DAPSA28, Disease Activity index for PSoriatic Arthritis in 28 joints; HAQ, Health Assessment Questionnaire; PGA, Patient Global Assessment; SJC swollen joint count; TJC tender joint count. | | | | | | | | |

**Supplementary Table S4 continued. Baseline characteristics of patients according to the presence or absence of each cardiometabolic comorbidity**

|  | Ischemic heart disease | | | |
| --- | --- | --- | --- | --- |
|  | **Absent (N=5,178)** | | **Present (N=121)** | |
|  | **Results** | **Availability** | **Results** | **Availability** |
| Age, years | 55 (46, 63) | 100% | 66 (60, 74) | 100% |
| Sex, female | 2,744 (53%) | 100% | 38 (31%) | 100% |
| BMI, kg/m^2^ | 27.5 (24.5, 31.4) | 83% | 29.0 (26.0, 32.4) | 87% |
| Smoking status |  |  |  |  |
| Never | 2,341 (54%) | 83% | 50 (48%) | 87% |
| Former | 1,069 (25%) |  | 37 (35%) |  |
| Current | 905 (21%) |  | 18 (17%) |  |
| Years since diagnosis | 9 (6, 14) | 66% | 12 (7, 19) | 94% |
| *Comorbidities* | | | | |
| Obesity | 967 (19%) | 100% | 38 (31%) | 100% |
| Dyslipidemia | 448 (8.7%) | 100% | 55 (45%) | 100% |
| Diabetes | 320 (6.2%) | 100% | 29 (24%) | 100% |
| Hypertension | 1,081 (21%) | 100% | 87 (72%) | 100% |
| Ischemic heart disease | 0 (0%) | 100% | 121 (100%) | 100% |
| *Disease activity* | | | | |
| CRP, mg/L | 5 (2, 13) | 98% | 5 (2, 11) | 88% |
| 28 SJC | 3 (0, 6) | 78% | 3 (0, 7) | 85% |
| 28 TJC | 4 (1, 10) | 78% | 5 (2, 12) | 85% |
| DAPSA28 | 20 (13, 31) | 41% | 23 (14, 31) | 46% |
| PGA, 0-10 mm | 6 (4, 7) | 51% | 6 (4, 8) | 54% |
| HAQ, 0-3 | 1.00 (0.50, 1.50) | 70% | 1.13 (0.50, 1.75) | 81% |
| *b/tsDMARD treatment start year* | | | | |
| 2015-2017 | 1,325 (26%) | 100% | 42 (35%) | 100% |
| 2018-2020 | 1,973 (38%) | 100% | 39 (32%) | 100% |
| 2021-2024 | 1,880 (36%) | 100% | 40 (33%) | 100% |
| Data are as observed, median (25%, 75% percentiles) unless otherwise stated. Abbreviations: BMI, Body Mass Index; CRP, C-reactive protein; DAPSA28, Disease Activity index for PSoriatic Arthritis in 28 joints; HAQ, Health Assessment Questionnaire; PGA, Patient Global Assessment; SJC swollen joint count; TJC tender joint count. | | | | |

**Supplementary Table S5 Prescription patterns of b/tsDMARD according to comorbidity burden in each individual registry**

|  | **bDMARD** | | | **tsDMARD** | |
| --- | --- | --- | --- | --- | --- |
|  | **TNFi** | **IL-17i** | **IL-12/23i or**  **IL-23i** | **JAKi** | **PDE4i** |
| **ATTRA, Czech Republic (n=1418)** | **1145, (81%)** | **273, (19%)** | **0, (0%)** | **0, (0%)** | **0, (0%)** |
| No comorbidity (n=561) | 447, (80%) | 114, (20%) | 0, (0%) | 0, (0%) | 0, (0%) |
| One comorbidity (n=409) | 336, (82%) | 73, (18%) | 0, (0%) | 0, (0%) | 0, (0%) |
| Two or more comorbidities (n=448) | 362, (81%) | 86, (19%) | 0, (0%) | 0, (0%) | 0, (0%) |
|  | | | | | |
| **BIOBADASER, Spain (n=109)** | **86, (78%)** | **12, (11%)** | **3, (3%)** | **2, (2%)** | **6, (6%)** |
| No comorbidity (n=95) | 74, (78%) | 12, (13%) | 3, (3%) | 0, (0%) | 6, (6%) |
| One comorbidity (n=10) | 9, (90%) | 0, (0%) | 0, (0%) | 1, (10%) | 0, (0%) |
| Two or more comorbidities (n=4) | 3, (75%) | 0, (0%) | 0, (0%) | 1, (25%) | 0, (0%) |
|  | | | | | |
| **BIORX.SI, Slovenia (n=360)** | **302, (84%)** | **41, (11%)** | **9, (3%)** | **1, (<1%)** | **7, (2%)** |
| No comorbidity (n=194) | 162, (84%) | 24, (12%) | 5, (3%) | 0, (0%) | 3, (2%) |
| One comorbidity (n=95) | 83, (87%) | 8, (8%) | 3, (3%) | 0, (0%) | 1, (1%) |
| Two or more comorbidities (n=71) | 57, (80%) | 9, (13%) | 1, (1%) | 1, (1%) | 3, (4%) |
|  | | | | | |
| **NOR-DMARD, Norway (n=237)** | **234, (99%)** | **3, (1%)** | **0, (0%)** | **0, (0%)** | **0, (0%)** |
| No comorbidity (n=179) | 178, (99%) | 1, (1%) | 0, (0%) | 0, (0%) | 0, (0%) |
| One comorbidity (n=41) | 39, (95%) | 2, (5%) | 0, (0%) | 0, (0%) | 0, (0%) |
| Two or more comorbidities (n=17) | 17, (100%) | 0, (0%) | 0, (0%) | 0, (0%) | 0, (0%) |
|  | | | | | |
| **RABBIT-SpA, Germany (n=830)** | **459, (55%)** | **236, (29%)** | **44, (5%)** | **25, (3%)** | **66, (8%)** |
| No comorbidity (n=425) | 257, (60%) | 115, (27%) | 16, (4%) | 9, (2%) | 28, (7%) |
| One comorbidity (n=235) | 112, (48%) | 69, (29%) | 21, (9%) | 11, (5%) | 22, (9%) |
| Two or more comorbidities (n=170) | 90, (53%) | 52, (31%) | 7, (4%) | 5, (3%) | 16, (9%) |
|  | | | | | |
| **Reuma.pt, Portugal (n=1207)** | **1012, (84%)** | **121, (10%)** | **35, (3%)** | **39, (3%)** | **0, (0%)** |
| No comorbidity (n=1013) | 850, (84%) | 100, (10%) | 25, (2%) | 38, (4%) | 0, (0%) |
| One comorbidity (n=118) | 96, (81%) | 15, (13%) | 6, (5%) | 1, (1%) | 0, (0%) |
| Two or more comorbidities (n=76) | 66, (87%) | 6, (8%) | 4, (5%) | 0, (0%) | 0, (0%) |
|  | | | | | |
| **ROBFIN, Finland (n=806)** | **571, (71%)** | **60, (7%)** | **19, (2%)** | **28, (4%)** | **128, (16%)** |
| No comorbidity (n=665) | 486, (73%) | 44, (7%) | 15, (2%) | 26, (4%) | 94, (14%) |
| One comorbidity (n=92) | 55, (60%) | 12, (13%) | 3, (3%) | 2, (2%) | 20, (22%) |
| Two or more comorbidities (n=49) | 30, (61%) | 4, (8%) | 1, (2%) | 0, (0%) | 14, (29%) |
|  | | | | | |
| **SCQM, Switzerland (n=332)** | **193, (58%)** | **53, (16%)** | **12, (4%)** | **6, (2%)** | **68, (20%)** |
| No comorbidity (n= 271) | 163, (60%) | 42, (16%) | 10, (4%) | 4, (1%) | 52 (19%) |
| One comorbidity (n=56) | 28, (50%) | 10, (18%) | 2, (4%) | 2, (4%) | 13, (23%) |
| Two or more comorbidities (n=6) | 2 (33%) | 1, (17%) | 0, (0%) | 0, (0%) | 3, (50%) |
| Row percentages of number of patients are presented, where the distribution of b/tsDMARDs sums to 100%. bDMARD, biologic disease-modifying anti-rheumatic drug; csDMARD, conventional synthetic disease-modifying anti-rheumatic drug; i, inhibitor; IL, interleukin; JAKi, Januskinase inhibitor; PDE4i, Phosphodiesterase 4 inhibitor; TNFi, Tumor Necrosis Factor inhibitor; tsDMARD, targeted synthetic disease-modifying anti-rheumatic drug. | | | | | |
